# Advances in the Treatment of Anal Fistula: A Mini-Review of Recent Five-Year Clinical Studies

**DOI:** 10.3389/fsurg.2020.586891

**Published:** 2021-02-11

**Authors:** Lijiang Ji, Yang Zhang, Liang Xu, Jun Wei, Liping Weng, Jie Jiang

**Affiliations:** ^1^Department of Anorectal Surgery, Changshu Hospital Affiliated to Nanjing University of Chinese Medicine, Changshu, China; ^2^Colorectal Disease Center, Nanjing Hospital of Chinese Medicine Affiliated to Nanjing University of Chinese Medicine, Nanjing, China; ^3^Department of General Surgery, Changshu Hospital Affiliated to Nanjing University of Chinese Medicine, Changshu, China

**Keywords:** anal fistula, sphincter-sparing, Seton, ligation of the intersphincteric fistula tract, healing rate, complications, recurrence rate

## Abstract

Anal fistula, with its complicated pathogenesis, has been considered as a clinical challenge for centuries. The risk of frequent recurrence and incontinence constitutes a considerable threat in the long-term treatment of anal fistula. In this work, we narratively reviewed the scientific literature of new techniques that have been used for anal fistula treatment over the recent 5 years, objectively evaluated the pros and cons of each technique on the basis of clinical outcomes, and tried to disclose the effective strategies for anal fistula treatment. Up to date, surgery is the main method used for treating anal fistula, but there is no simple technique that can completely heal complex anal fistula. In the course of surgery treatment, the healing outcome, and the protection of anal function should be weighed comprehensively. Among the innovative techniques that have emerged in recent years, combined techniques based on drainage Seton and LIFT-plug seem to be the relatively effective therapies, but their effectiveness requires more multi-center prospective randomized controlled trials with large sample size and long-term follow-up to be validated.

## Introduction

Anal fistula is a sequela of the abscess ulceration or incision drainage that occurs around the anus and rectum, which is manifested as the formation of abnormal channels connecting the anal canal and rectum with the skin around the anus. There are 20,000 to 25,000 newly confirmed cases in the USA each year (1). A statistical analysis based on a large population database in the UK showed that the incidence of anal fistula is 1.69 cases per 10,000 individuals (2). This was also evidenced by other relevant studies (3). Patients with anal fistula are mainly adults between 30 and 40 years old, and the incidence rate of this condition in men is higher than that in women (4). In addition to severely affecting the quality of life of patients, anal fistula has also a negative impact on the psychological state of patients who often suffer from depression or anxiety symptoms. In general, anal fistula cannot be cured without therapeutic intervention. Surgical therapy is the main method used to treat anal fistula. The best treatment criterion is to eradicate the infected lesion, ensure sufficient drainage, and promote the closure of the fistula, while minimizing damage to the anal sphincter (5). The integrity of the internal anal sphincter (IAS) and external anal sphincter (EAS) is the most important guarantee for keeping normal anal function of patients.

Anal fistula can be divided into simple and complex types according to the degree of lesions. According to the classification standards of the American Society of Colon and Rectal Surgeons (ASCRS), the former includes low transphincteric, and intersphincteric fistulas, which account for lesser than 30% of the sphincter complex. Regarding simple anal fistula, especially distal cases, fistulotomy can be used to obtain ideal treatment results (6). However, complex anal fistula is one of the refractory diseases encountered in colorectal surgery; it is transphincteric fistula that account for more than 30% of sphincter complex, and includes anal fistulas related to malignancy, inflammatory bowel disease, radiation, chronic diarrhea, or preexisting fecal incontinence. Due to the diverse causes and forms of complex anal fistula, its treatments are often accompanied by a high risk of recurrence and potential incontinence disorders, and there still is a lack of clinical consensus on the best surgical approach. Cutting Seton is a preliminary exploration of sphincter-sparing technology. It works on the principle that gradual detachment of muscles will lead to fibrosis and necrosis, which can maintain the integrity of the sphincter complex with minimal damage to the cutting end (5). Nevertheless, studies have shown that cutting Seton does not sufficiently protect the anal sphincter (7, 8), and the postoperative anal incontinence rate was even as high as 63% (9). The slow section of the sphincters by cutting Seton produces a sphincter injury, with outcomes perhaps even less controllable than a simple lay-open fistulotomy. Total sphincter preservation surgery has gradually become the first choice for anal fistula treatment. In the past few decades, several sphincter-sparing techniques have been established, including endorectal advancement flap (ERAF), ligation of the intersphincteric fistula tract (LIFT), fibrin glue, anal fistula plug, fistula laser closure, video-assisted anal fistula treatment (VAAFT), and adipose-derived stem cells; the last review of these studies was published in 2015 (10). Based on these independent sphincter-sparing techniques, to further diminish the recurrence rate, protect the anal sphincter and obtain better postoperative outcomes, some innovative, combined, and modified new therapies have been proposed and applied in clinical studies in recent years. However, due to the diversity of treatment methods and the inevitable heterogeneity of clinical trials, their variable outcomes are prone to generate confusion and misunderstanding.

Therefore, this review aimed to systematically summarize the new anal fistula therapies that have been applied in clinics in the past 5 years, and critically evaluate these methods from the perspective of healing rate, complication, and recurrence rate in order to assess the feasibility of these anal fistula treatment techniques.

## Methodology

A mini-review of the last 5-year (January 2015 to September 2020) studies focused on the treatment of anal fistula was performed using papers obtained from electronic databases, including PubMed, Web of science, Embase, and Cochrane library. The search was restricted to articles published in English language and the search terms included “anal fistula,” “fistula-in-ano,” “treatment,” and “sphincter-sparing.” Finally, 29 papers were selected, which included 21 prospective cohort studies (11–31), seven retrospective cohort studies (32–37), and one case report (38).

### Advanced Techniques

#### Modified Seton

Taking into account the insufficient anal sphincter protection during the treatment of anal fistula by cutting Seton, a preliminary improvement method of drainage Seton (loose Seton) was proposed, which forms continuous drainage of the fistula through medical thread, rubber band, and other materials to prevent the formation of abscess. Although the drainage Seton completely preserves the sphincter and reduces anal incontinence, previous study results showed that the long-term recurrence rate for complex anal fistula treatment was up to 20–80% (39, 40). Haennig et al. (12) performed a prospective observational cohort study involving 81 patients with Crohn's disease-induced perianal fistula to evaluate the treatment effectiveness of drainage Seton combined with infliximab. It was found that fistulas in 72.5% of patients were closed, 13.6% of patients had acute adverse events related to anti-TNFα therapy, and the recurrence rate was 45.8% after a long-term follow-up (Table 1). Among them, gender, rectum-vaginal, anal stenosis, and complex fistula were considered to be the association factors of treatment failure. Izadpanah et al. (16) introduced another combination therapy called pulling Seton based on Seton technique, which combined non-cutting and cutting mechanisms. As listed in the Table 1, this prospective study showed that 94% of 201 patients with high anal fistula were healed, and the incontinence rate and recurrence rate were controlled within a small and acceptable range (<5%) (16). In addition, the concept of rerouting of the fistula tract was first proposed by Mann and Clifton (41). This technique can effectively treat anorectal fistula and high anal fistula by relocating the fistula of the sphincteric external part to the position between the sphincter and immediately repairing the EAS. Omar et al. (28) implemented a prospective randomized controlled trial to compare the clinical outcomes between the separate drainage Seton and drainage Seton combined with fistula tract rerouting (EAS-sparing Seton after rerouting) and their results showed that there was no significant difference in the recurrence rate and complications between the two groups, but the combined technique reduced the postoperative healing time and the number of patients who required secondary fistulotomy. Abdelnaby et al. (24) further combined the drainage Seton, fistula tract rerouting, and mucosal advancement flap. They found that this triple therapy could significantly reduce fecal incontinence, but it took more time for surgery and recovery when compared with the method of rerouting Seton around the IAS. In short, after gradual improvement, Seton-based techniques such as pulling Seton, EAS-sparing Seton after rerouting, and rerouting Seton around the EAS combined with mucosal advancement flap have become more efficient methods for high anal fistula treatment compared to the conventional seton method.

**Table 1:** Published articles on new techniques of modified Seton for anal fistula treatment in the past 5 years.

**Studies**	**Type of study**	**Advanced techniques**	**No. patients**	**Follow-up (week)**	**Healing rate (%)**	**Complications (%)**	**Recurrence rate (%)**
Abdelnaby et al. (24)	Prospective randomized controlled study	Rerouting Seton around the EAS combined with mucosal advancement flap	49	82.8 ± 17.2	95.9	8.2	4.1
		Rerouting Seton around the IAS	48	82 ± 17.6	91.7	6.3	8.3
Haennig et al. (12)	Prospective observational cohort study	Drainage Seton combined with anti-TNFα therapy	81	256 (8–1,052)	72.5	13.6	45.8
Izadpanah et al. (16)	Prospective observational cohort study	Pulling Seton	201	264 (96–384)	94.0	3.0	4.5
Omar et al. (28)	Prospective randomized controlled study	Drainage Seton	30	48 ± 12	87.0	17.0	13.0
		EAS-sparing Seton after rerouting	30	48 ± 12	97.0	7.0	3.0

*IAS, internal anal sphincter; EAS, external anal sphincter*.

#### Modified LIFT

LIFT is an effective and low-cost sphincter-sparing technique which was first introduced by Rojanasakul et al. (42), with a success rate of 94.4% and the absence of continence failures (42). LIFT technique mainly ligates and cuts the fistula between the sphincters, scrapes the infected tissue of the fistula wall, and tightens the fistula tract with ligation, which can effectively avoid repeated infections caused by fecal particles. It is appropriate for transsphincteric fistulas that have well-formed fistulas including most complex anal fistulas (43), recurrent anal fistula (44), and fistulas that fail after other surgical procedures, but not for early fistulas without clearly formed fistula. Additionally, another limitation of LIFT is that the healing outcome is unstable; some previous studies have shown that the success rate is only about 50% (44, 45). Emile et al. (46) suggested that Crohn's disease, horseshoe fistulas, and previous fistula surgery are predictors of LIFT failure. A report published in 2013 tracked the long-term results after LIFT treatment, and concluded that long anal tracts were a part of the many postoperative failures (47). To improve the healing rate of LIFT technique, “medialization of the external opening to the intersphincteric wound” was suggested as one of the corrective measures in LIFT failure cases (48). Wright et al. (49) indicated that the placement of Seton followed by fistulotomy and rectal advancement flap could treat 50% of the patients with LIFT failures.

In recent years, LIFT technique has gained in popularity worldwide; some improved techniques based on LIFT have also been proposed. For instance, Arroyo et al. (19) improved the LIFT technique and proved that ligation of the intersphincteric fistula, but not excision, was safe and effective for treating anal fistula via a prospective observational trial (Table 2). Another modified LIFT technique was carried out by Kang et al. (23), who performed LIFT by lateral approach which cuts along the fistula from the external opening until the sphincter space is exposed, and ligation is undertaken near the internal anal sphincter followed by the removal of the ligated distal part. However, the above two methods have relatively high recurrence rates, 21 and 18%, respectively (Table 2). In 2016, a multi-center prospective randomized trial demonstrated that when compared to the simple LIFT technique, the LIFT-plug method, which is an improved LIFT procedure by addition of a bioprosthetic anal fistula plug, increased the healing rate from 83.9 to 94% and shortened the time required for healing (15). Similarly, Zhao et al. (35) found LIFT-plug procedure as a convenient method for treating transsphincteric perianal fistulas via a long-term retrospective cohort study (Table 2). Their results showed that LIFT-plug procedure led to a high healing rate, low trauma, and effective protection of anal sphincter. Another modification in LIFT procedure proposed by Zwiep et al. (37) recommended placing a “biological mesh” on the plane between the sphincters (BioLIFT). This retrospective cohort study showed that the success rate and safety of this technique was reasonable, but the cost factor was one of its limitations (37). Based on these studies, we concluded that LIFT-plug and BioLIFT were two alternative solutions for the treatment of transsphincteric anal fistula. However, the improved advantages of these new techniques need more prospective studies for further confirmation.

**Table 2:** Published articles on new techniques of modified LIFT for anal fistula treatment in the past 5 years.

**Studies**	**Type of study**	**Advanced techniques**	**No. patients**	**Follow-up (week)**	**Healing rate (%)**	**Complications**	**Recurrence rate (%)**
Araújo et al. (18)	Prospective observational cohort study	LIFT without excision of the fistula tract	38	32 (14-56)	79.0	0.0%	21.0
Han et al. (15)	Prospective randomized controlled study	LIFT	118	24	83.9	0.0%	0.0
		LIFT-plug	117	24	94.0	0.0%	0.0
Kang et al. (23)	Prospective observational cohort study	LIFT by lateral approach	28	64(32–108)	75.0	0.0%	18.0
Zhao et al. (35)	Retrospective cohort study	LIFT-plug	78	120 (64–188)	96.2	6.7%	2.7
Zwiep et al. (37)	Retrospective cohort study	LIFT	75	29	58.7	17.3%	41.3
		BioLIFT	44	9	75.0	22.7%	25.0

*LIFT, ligation of the intersphincteric fistula tract*.

### OTSC® (Over-the-Scope-Clip) Proctology Device

OTSC® proctology device is a system of elastic nitinol alloy closure clip, and is used to close the fistula tract from inside by placing the device on the internal opening of fistula. Prosst et al. (50) first applied the experience of gastrointestinal endoscopy to the treatment of anal fistula by modifying the proctology clip in 2012. Recently, a 3-year long-term retrospective study showed that the fistula tracts were completely closed in 59% of 22 patients with anal fistula (51). Similarly, the results of a retrospective cohort study which enrolled 10 patients treated with the OTSC® proctology device indicated that the nitinol closure clip had to be removed from one patient due to persistent anal pain; three of nine recovered patients had relapsed during a short-term follow-up, and all patients had no complication of postoperative fecal incontinence (32). In addition, a prospective randomized controlled study carried out by Mascagni et al. (27) showed that 93.3% of 15 anal fistula patients treated with OTSC® proctology device were healed (Table 3), and the clinical outcomes of OTSC® proctology device treatment were better than those of fistulectomy and primary sphincteroplasty (FIPS; described below) in many aspects, including the postoperative pain, anal sphincter protection, recovery time, and length of hospital stay. As shown in Table 3, although the procedure was characterized by tolerability and minimal invasiveness, the success rate of OTSC clip fluctuated and the recurrence rate was high in the two retrospective analyses of small sample sizes (32, 51). Therefore, the effectiveness of OTSC® proctology device needs additional validation with a large-scale prospective randomized trial.

**Table 3:** Published articles on new techniques of OTSC®proctology device for anal fistula treatment in the past 5 years.

**Studies**	**Type of study**	**Advanced techniques**	**No. patients**	**Follow-up (week)**	**Healing rate (%)**	**Complications**	**Recurrence rate (%)**
Dango et al. (51)	Retrospective cohort study	OTSC®proctology device	22	144 (76–192)	59.0	0.0%	41.0
Marinello et al. (32)	Retrospective cohort study	OTSC®proctology device	10	60 (24–104)	60.0	0.0%	33.3
Mascagni et al. (27)	Prospective randomized controlled study	OTSC®proctology device	15	144	93.3	0.0%	6.7

*OTSC, over the scope clip*.

### FIPS

Even though fistulotomy is still the preferred technique for simple anal fistula treatment, high incontinence rate and keyhole deformity are the two main drawbacks of the traditional fistulotomy. The sphincter-sparing method has been widely recognized in the recent decades, but the high recurrence rate remains an important challenge for colorectal surgeons. In fact, as early as 1985, Parkash et al. (52) proposed an immediate sphincter repair technique to improve fistulotomy, namely FIPS. Compared with the conventional fistulotomy, FIPS reduces the risk of postoperative keyhole deformity and fecal incontinence. Moreover, compared with the majority of sphincter preservation techniques, FIPS decreases the recurrence rate after surgery. Therefore, this compromised method has received new attention in recent years (Table 4).

**Table 4:** Published articles on new techniques of FIPS for anal fistula treatment in the past 5 years.

**Studies**	**Type of study**	**Advanced techniques**	**No. patients**	**Follow-up (week)**	**Healing rate (%)**	**Complications**	**Recurrence rate (%)**
De et al. (36)	Retrospective cohort study	FIPS	24	12 (8–25.2)	95.8	25.0%	4.2
Litta et al. (34)	Retrospective cohort study	FIPS	203	224 ± 124	93.0	13.0%	8.0
Mascagni et al. (27)	Prospective randomized controlled study	FIPS	15	144	100.0	0.0%	0.0
Seyfried et al. (33)	Retrospective cohort study	FIPS	424	44 (28–800)	88.2	23.0%	4.2

*FIPS, fistulectomy and primary sphincteroplasty*.

A retrospective clinical trial designed by De Hous et al. (36) proved that FIPS could avoid the formation of keyhole deformity in the treatment of patients with simple anal fistula and pointed out that the occurrence of keyhole deformity was positively correlated with posterior fistula. Besides, the postoperative recurrence rate of FIPS was within an acceptable range (4.2%). In addition, the retrospective evaluation of Seyfried et al. (33) found that primary sphincter reconstruction not only had satisfactory effects on the distal fistula, but also an effective treatment for intermediate fistula, and even led to comparable clinical outcomes in the treatment of proximal fistula. However, the therapeutic effect of this technique decreased with the complexity of anal fistula. Litta et al. (34) also confirmed that patients with complex or transsphincteric anal fistula had lower postoperative satisfaction with FIPS. From these findings, it was revealed that FIPS is a simple, efficient, and low-recurrence anal fistula therapy, especially for simple anal fistula, but the potential risk of incontinence and keyhole deformity after surgery should be informed to patients before surgery.

### Filling Therapy

In recent years, some derivative methods based on anal fistula plug and fibrin glue have also been proposed and developed. These methods are mainly improved by optimizing the synthetic material of the implanted fistula plug (Table 5). For example, Bobkiewicz et al. (38) introduced a new technique to treat anal fistula by establishing a novel biomaterial model for making anal fistula plug. They found that the plug made from acellular dermal matrix could fuse with the tissue and proliferate excessively in the fistula. Additionally, the operation process was simple, and the operation time and postoperative recovery time were short. However, this study was a case report and lacked long-term clinical trial verification with a large sample size. Similarly, other filling matrices, including platelet-rich plasma (PRP), autologous cartilage, fat, autologous micro-fragmented adipose tissue, and allogeneic bone marrow-derived mesenchymal stromal cells (MSCs), have also been reported for the treatment of anal fistula. In their work, de la Portilla et al. (25) implemented a prospective double-blind randomized trial to compare the effectiveness of autologous PRP and fibrin glue in cryptogenic anal fistula treatment. The results showed that the therapeutic effects of these two techniques were equivalent and there was no incontinence-related adverse events, but their recurrence rates were high, 33.3 and 31.3%, respectively (Table 5). Ozturk (14) used autologous cartilage as a raw material for anal fistula plug. A 2-year follow-up revealed that the fistula tracts in 90% of the 10 patients were closed after surgery, and there were no short-term complications, but two of the recovered patients relapsed in the long-term follow-up. Recently, autologous fat tissues have been considered as safe, effective, and feasible biomaterials in the treatments of anal fistula. Stroumza et al. (22) evaluated the efficacy of fat transplantation in the treatment of anal fistula through a prospective observational trial. The results showed that 73% of patients recovered completely within 6-month follow-up without any side effect (Table 5). A similar prospective clinical trial designed by Laureti et al. (30) proved that autologous micro-fragmented adipose tissue injection allowed the recovery of 66.7% of patients after surgery, and there was no complication recorded. Moreover, through a prospective double-blind placebo-controlled trial, Molendijk et al. (13) found that bone MSCs from healthy donors can effectively treat perianal fistula induced by Crohn's disease compared with placebo treatment, and the efficacy was related to the cell density of bone MSCs; the effect was best when the cell concentration was 3 × 10^7^ (Table 5). Furthermore, Ratto et al. (17) initially explored the effectiveness of Curaseal AF^™^ device in anal fistula treatment through a short-term (6 months) clinical trial; their results indicated that the technique was simple, effective, and did not increase the risk of postoperative incontinence, but these preliminary results require further validation by more clinical trials with longer follow-up and larger sample size. In summary, these multivariate filling matrices made of biomaterials are feasible therapies for anal fistula treatment. The common advantages of their clinical outcomes are the complete preservation of the anal sphincter and zero side effect. The primary disadvantage is that the recurrence rate is relatively high, especially for the long-term recurrence rate.

**Table 5:** Published articles on new techniques of filling therapy for anal fistula treatment in the past 5 years.

**Studies**	**Type of study**	**Advanced techniques**	**No. patients**	**Follow-up (week)**	**Healing rate (%)**	**Complications (%)**	**Recurrence rate (%)**
Bobkiewicz et al. (38)	Case report	Acellular dermal matrix plug	1	8	100.0	0.0	0.0
de et al. (25)	Prospective randomized controlled study	PRP	32	48	71.0	0.0	33.3
		Fibrin glue	24	48	58.3	0.0	31.3
Ozturk (14)	Prospective observational cohort study	Autologous cartilage plug	10	96 (40-128)	70.0	0.0	20.0
Stroumza et al. (22)	Prospective observational cohort study	Fat GRAFT technique	11	24	73.0	0.0	27.0
Laureti et al. (30)	Prospective observational cohort study	Autologous micro-fragmented adipose tissue	15	24	66.7	0.0	0.0
Molendijk et al. (13)	Prospective randomized, placebo-controlled study	Allogeneic bone MSCs 1 × 10^7^	5	24	80.0	0.0	0.0
		Allogeneic bone MSCs 3 × 10^7^	5	24	80.0	0.0	0.0
		Allogeneic bone MSCs 9 × 10^7^	5	24	20.0	0.0	0.0
		Placebo	6	24	33.3	0.0	50.0
Ratto et al. (17)	Prospective observational cohort study	Curaseal AF^™^ device	10	24	70.0	0.0	30.0

*PRP, platelet-rich plasma; GRAFT, grafting in anal fistula treatment; MSCs, marrow-derived mesenchymal stromal cells*.

### Photodynamic Therapy (PDT)

In 2017, Arroyo et al. (19) first introduced the PDT to the anal fistula treatment. PDT is a treatment that combines light energy and photosensitizers to induce photooxidative damage to target tissues or cells. It has been mainly used in cancer treatment before. Through two long-term prospective observational trials, Arroyo et al. (19) and Arroyo et al. (29) showed that PDT was an effective sphincter-sparing therapy with a simple surgical procedure, high safety, and healing rate ranging from 65.3 to 80% (Table 6), which can be considered as an alternative choice for patients with complex anal fistula. However, to date, clinical research related to PDT is still very limited, and the cost of this technique is higher than that of traditional surgery; thus, practitioners need to carefully evaluate its cost-effectiveness when choosing treatment options.

**Table 6:** Published articles on new techniques of PTD for anal fistula treatment in the past 5 years.

**Studies**	**Type of study**	**Advanced techniques**	**No. patients**	**Follow-up (week)**	**Healing rate (%)**	**Complications (%)**	**Recurrence rate (%)**
Arroyo et al. (19)	Prospective observational cohort study	PDT	10	59.6(48–80)	80.0	0.0	20.0
Arroyo et al. (29)	Prospective observational cohort study	PDT	49	48	65.3	6.3	34.7

*PDT, photodynamic therapy*.

### Other Surgical Procedures

In addition to the six types of treatments described above, as listed in the Table 7, some other surgical treatments for anal fistulas such as proximal superficial cauterization, emptying regularly fistula tracts and curettage of tracts (PERFACT) procedure, transanal opening of intersphincteric space (TROPIS), and tunnel-like fistulectomy plus draining Seton combined with incision of internal opening of anal fistula (TFSIA) have also been proposed. In 2015, a new procedure called PERFACT for high complex anal fistula treatment was proposed. The key of this technique is to cauterize the mucosal surfaces located around the internal opening of the fistula, and to preserve the tracts (fistulas, branches, and cavities) clean (11). Their prospective cohort trial proved that 79.5% of the patients were effectively treated and there was no case of surgery-induced incontinence being recorded (Table 7). Two years later, the same authors reported another new technique for the same disease, called TROPIS (20). The results showed that TROPIS technique increased the healing rate to 84.5%, and the overall healing rate exceeded 90%. In addition, a prospective randomized controlled trial conducted by Yan and Ma (31) found that although the clinical outcomes of TFSIA and cutting Seton did not differ between the healing rate and the recurrence rate, the former significantly reduced the postoperative recovery time, keyhole deformity rate, and incontinence score.

**Table 7:** Published articles on new techniques of other surgical procedures for anal fistula treatment in the past 5 years.

**Studies**	**Type of study**	**Advanced techniques**	**No. patients**	**Follow-up (week)**	**Healing rate (%)**	**Complications**	**Recurrence rate (%)**
Garg and Garg (11)	Prospective observational cohort study	PERFACT procedure	44	36 (20–56)	79.5	9.1%	20.5
Garg (20)	Prospective observational cohort study	TROPIS	52	36(24–84)	84.6	1.9%	15.4
Yan and Ma (31)	Prospective randomized controlled study	TFSIA	40	24	87.5	0.7 ± 0.5^#^	12.5
		Cutting Seton	40	24	85.0	1.2 ± 0.7^#^	15.0

PERFACT, proximal superficial cauterization, emptying regularly fistula tracts, and curettage of tracts; TROPIS, transanal opening of intersphincteric space; TFSIA, tunnel-like fistulectomy plus draining Seton combined with incision of internal opening of anal fistula;

# *Wexner anal incontinence score after 6 months of operation*.

### Non-surgical Procedures

Since 2015, some non-surgical methods for the treatment of anal fistula have gradually emerged as well, but the functions of these methods are often limited and even ineffective, and they are not satisfactory alternative methods for anal fistula surgery (Table 8). For instance, Iqbal et al. (26) proposed a non-surgical therapy for the treatment of low perianal fistula by washing fistula with 1% silver nitrate solution. Their prospective observational trial proved that silver nitrate successfully healed 76.3% of patients with low perianal fistula, indicating that this simple, easily realized, and side-effect-free therapy was feasible for the treatment of low perianal fistula (Table 8). Additionally, a previous study tried to use ozone to treat chronic anal fistula (21). Although the treatment did not have any side effect or complication induced by ozone injection, the cure rate was only 25% among the 12 patients who participated in the prospective clinical trial, and one third of the recovered patient relapsed, so this technique has not yet been conventionally recognized.

**Table 8:** Published articles on new techniques of non-surgical procedures for anal fistula treatment in the past 5 years.

**Studies**	**Type of study**	**Advanced techniques**	**No. patients**	**Follow-up (week)**	**Healing rate (%)**	**Complications (%)**	**Recurrence rate (%)**
Iqbal et al. (26)	Prospective observational cohort study	1% silver nitrate	76	10	76.3	0.0	23.7
Ozturk et al. (21)	Prospective observational cohort study	Ozone	12	48	25.0	0.0	33.3

## Conclusion

With the increasing attention being paid to anorectal diseases and the continuous advancement of science and technology, a large number of new therapies have emerged to address the issue of complex anal fistula. Due to the characteristics of multiple inducements and forms of complex anal fistula, there is a lack of unified criteria for the efficacy evaluation of various anal fistula treatment methods, and no consensus has been reached on the best therapy. The specific implementation of a given treatment often has to formulate personalized schemes according to the actual situation of the patient. There is no simple technique that can completely heal complex anal fistulas. The effectiveness of surgery and the protection of anal function must be considered comprehensively during the treatment. As far as the current research results are concerned, several combined techniques based on drainage Seton (pulling Seton, EAS-sparing Seton after rerouting, and rerouting Seton around the EAS combined with mucosal advancement flap) and LIFT-plug are considered to be the feasible therapies of complex anal fistulas, but their clinical outcomes need more prospective randomized controlled trials with multi-center, large sample size, and long-term follow-up for credible confirmation. Additionally, due to the limited data available, which unfortunately is the nature of this research field, it is difficult to make reliable judgments about these technologies. So far, none of these treatment techniques have, however, added a paradigm shift in success rate when evaluated in larger and better controlled studies. In the future, a large number of high-quality clinical trials are urgently needed to jointly answer the question of the best strategy for anal fistula treatment.

## Author Contributions

LJ, YZ, LX, JW, LW, and JJ contributed in drafting the manuscript. LW conceptualized and designed the work. All authors contributed to the article and approved the submitted version.

## Conflict of Interest

The authors declare that the research was conducted in the absence of any commercial or financial relationships that could be construed as a potential conflict of interest.
